# New type of doping effect via metallization of surface reduction in SnO_2_

**DOI:** 10.1038/s41598-019-44634-0

**Published:** 2019-05-31

**Authors:** Jae Hoon Bang, Myung Sik Choi, Han Gil Na, Wansik Oum, Sun-Woo Choi, Sang Sub Kim, Hyoun Woo Kim, Changhyun Jin

**Affiliations:** 1 Division of Materials Science and Engineering, Hanyang University, Seoul, 04763 Republic of Korea; 2 Department of Materials Science and Engineering, Kangwon National University, Samcheok, 25913 Republic of Korea; 3 Department of Materials Science and Engineering, Inha University, Incheon, 402-751 Republic of Korea; 4 The Research Institute of Industrial Science, Hanyang University, Seoul, 04763 Republic of Korea

**Keywords:** Materials science, Nanoscale materials

## Abstract

The use of conventional doping methods requires consideration of not only the energy connection with the base material but also the limits of the type and doping range of the dopant. The scope of the physico-chemical change must be determined from the properties of the base material, and when this limit is exceeded, a large energy barrier must be formed between the base material and the dopant as in a heterojunction. Thus, starting from a different viewpoint, we introduce a so-called *metallization of surface reduction* method, which easily overcomes the disadvantages of existing methods while having the effect of doping the base material. Such new synthetic techniques enable sequential energy arrangements–gradients from the surface to the centre of the material–so that free energy transfer effects can be obtained as per the energies in the semiconducting band, eliminating the energy discontinuity of the heterojunction.

## Introduction

Even within a single material, the surface must physically and chemically differ from the interior due to its structural characteristic of being directly in contact with another medium. Therefore, since surfaces have unique physicochemical characteristics, a different approach to that used for bulk materials is required for methodological aspects of measuring the surface energy state. For example, (1) in the case of the calculation of surface free energy, the nearest-neighbour broken-bond (NNBB) model^1,2^ based on the hard-sphere model or Young’s modulus^3^ can be used because the surface itself has a crystallographically anisotropic structure. (2) For the surface structure, the nearest-neighbour bond model can be applied due to the slightly-misoriented plane caused by the terrace-ledge kink (TLK)^4,5^ at the surface. (3) For crystal growth on surfaces, diffusion-controlled^6^ and interface-controlled^7^ growth modes occur when a surface is rough and when a surface is a smooth (faceted) interface, respectively. Furthermore, only precise atomic-scale tools such as scanning probe microscopy (SPM), scanning tunnelling microscopy (STM), atomic force microscopy (AFM), magnetic force microscopy (MFM), and electrostatic force microscopy (EFM) may be used as measurement techniques^8–12^. Based on these considerations, research on material surfaces is underway across a wide range of fields. Indeed, one of the preferred methods of controlling the surface properties of materials, especially in terms of theoretical accessibility and efficiency, is via doping. However, as is well known, the Hume-Rothery rules^13^ require the following conditions to be satisfied for doping: for the two atoms, (1) the difference in radius must be within 15% and they must have (2) similar electronegativity, (3) the same crystal structure, and (4) similar valence electrons. Moreover, even if such considerations are fully satisfied, it is not easy to apply such stringent conditions in industrial settings due to the difficulty and cost of equipment installation, and the lack of accurate qualitative/quantitative control. Because of these practical difficulties, it is thought that creating doping effects by changing the constituents of the base material, rather than injecting atoms from outside, is more stable and facile. Further, considering the energy interaction, the density of the doping from the surface of the base material to its centre is thought to be gradated, so that the energy connections between the base material and the dopant are continuous. However, until now, doping has been a process of forcibly inserting the dopant into the base material from the outside and forcibly changing the carrier concentration inside the base material. Since this is not a chemical process inducing a natural reaction, it is assumed that it may be effective simply to physically increase the carrier, but the energy stability is relatively lower in the correlation between the base material and the dopant. Accordingly, in this study, we show that surface characteristics may be altered by a new surface metallization technique through reduction of a metal oxide, which we refer to as metallization of surface reduction (MSR). Our technique not only changes the carrier concentration of the base material, but also provides stability of the energy connection between the base material and the surface. Therefore, the application range is unlimited because the partial surface and entire base metal can be controlled freely and stably.

SnO_2_ is one of the typical n-type semiconducting oxides, and the main reason for this behaviour is that the stoichiometric 1:2 ratio of tin and oxygen is broken due to the presence of interstitial Sn atoms or the O vacancies^14,15^. Thus, theoretically, in a SnO_2_ compound, if the reduction reaction by desorption of O_2_ is activated, the effect may be to greatly increase the n-type electrical characteristics of SnO_2_ and consequently, such a reaction has the potential to improve the efficiency of many electronic devices. At present, to break the bond between tin and oxygen, different methodological approaches such as heat, electromagnetic waves, and ion beams may not be very important factors. In other words, the choice of which energy source to use in reducing SnO_2_ is one only of differences in accessibility, economics, and efficiency; however, the ultimate goal of such methods is the same. At this time, as the reduction reaction from the surface proceeds, the existing base material (SnO_2_) can act as a frame (or template) so that crystals can be formed without surface amorphousness, and sequential arrangement (i.e., SnO_2_-SnO_*x*_-Sn) from ceramic SnO_2_ (in the core) to metal Sn (at the surface) becomes possible.

**Figure 1:**
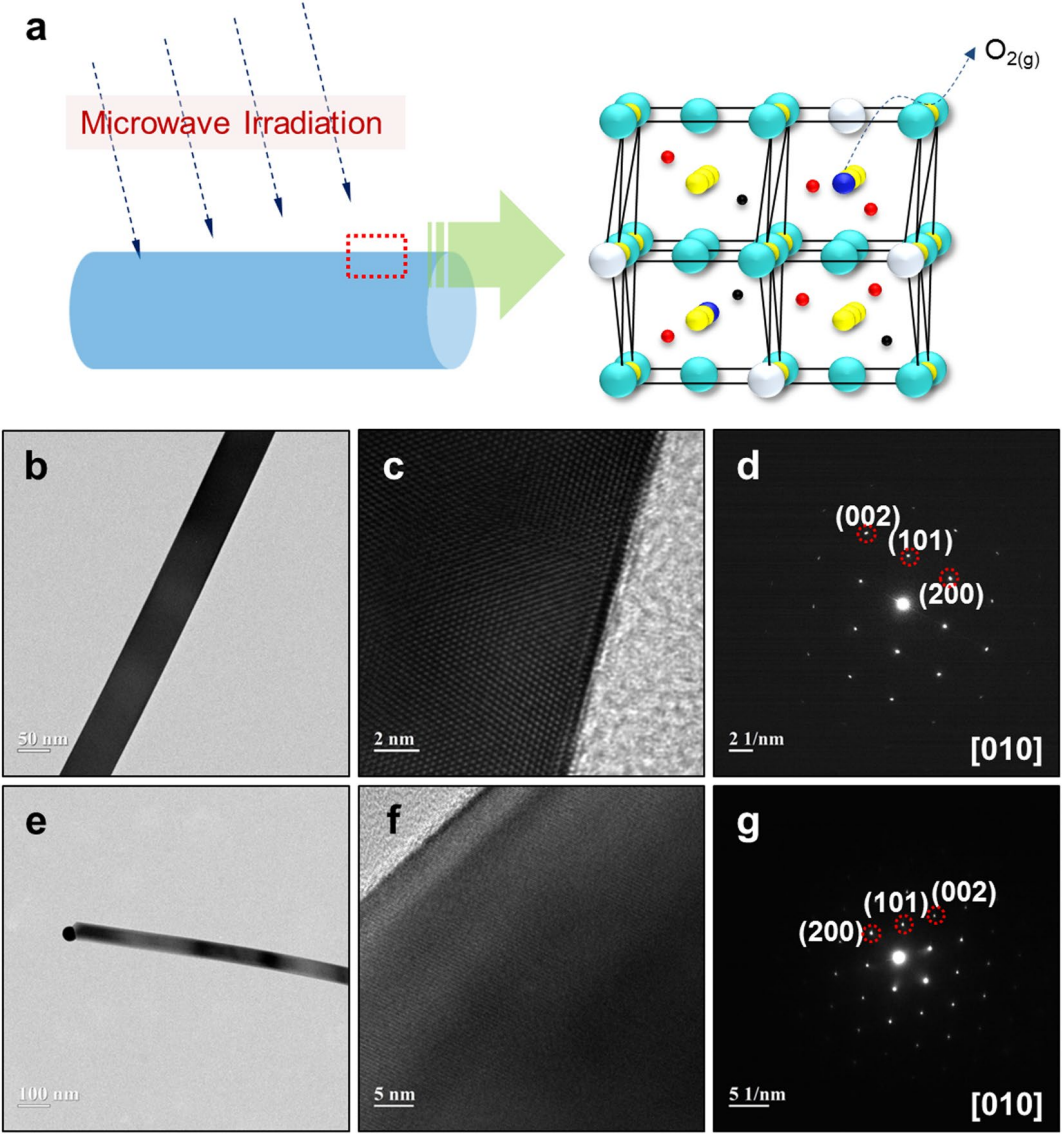
Oxygen vacancy formation with microwave energy in SnO_2_ nanowires and TEM imaging. (**a**) Schematic diagram showing the process of oxygen desorption and reduction to Sn on the surface of SnO_2_ nanowires (NWs) by microwave (MW) energy irradiation; (**b**–**g**) TEM images of SnO_2_ NWs (**b**–**d**) before and (**e**–**g**) after MW irradiation; (**b**,**e**) typical image of SnO_2_ NW; (**c**,**f**) high resolution TEM image; (**d**,**g**) SAED pattern representing single crystals. From above (**b**–**g**), the degree of oxidation on the SnO_2_ surface is difficult to distinguish by TEM measurement.

Figure 1a is a schematic diagram illustrating O_2_-desorbtion from the surface of a given SnO_2_ sample due to high energy microwave (MW) irradiation. At first glance, this concept shows two types of arrangements such as heterojunction^16–18^, and it can be considered that there is no difference compared to our samples. However, in the case of the heterojunction of Sn and SnO_2_, the contact between the metal and the semiconductor constrains energy transfer to occur because each material has a distinct energy potential. That is, since barriers associated with energy transfer can occur, a minimum energy to overcome is required, or else energy transfer becomes directional. This means that they do not represent a comprehensive characteristic as one but two independent characteristics; there is a clear boundary between the two materials within the heterojunction. Whereas, the mechanism of the generation of Sn via reduction of SnO_2_ using a high-energy source produces a gradient in the quantity of Sn, and a gradation is generated from the surface of SnO_2_ to its centre. This process of Sn-generation can be considered as forming a single material or a combination of various materials, depending on the analysis viewpoint. It can be regarded as a single material because it is difficult to distinguish clearly, but it can also be seen that various materials exist together because the composition varies continuously with depth from the surface to the centre, resembling the energy band structure^19,20^ formed by similar energy levels in a semiconductor. This energy band can be treated as a single entity in that the difference between the respective energy levels constituting it is so narrow that a smooth shift is possible even with a small energy difference. Therefore, it is clearly distinguished from an energy bandgap^21^ which is a noticeable energy difference between two energy bands. For this reason, it is not easy to detect the difference in the material because of the smooth and natural continuous energy difference, even if the surface portion is reduced due to high energy. Indeed, the morphological and crystallographic microstructure in the SnO_2_ nanowires (NWs) without MW irradiation (Fig. 1b–d) and with MW irradiation (Fig. 1e–g) did not show immediate obvious differences. Specifically, in both cases, the typical shapes of the SnO_2_ NWs were uniform in surface and a few tens of nanometres in diameter (Fig. 1b,e). Moreover, they have the regularity of single crystals (Fig. 1c,f) and this can be confirmed by the SAED pattern showing the (002), (101) and (200) planes obtained in the [010] direction (Fig. 1d,g)^22^. This suggests that even if the reduction reaction from SnO_2_ to Sn proceeds, it may be difficult to easily observe the physical and chemical properties of the product. To reconfirm this problem, both XRD data (Supplementary Fig. 1a,b)^23^ and TEM images (Supplementary Fig. 1c–e) acquired after irradiation with protons were recorded for comparison with the those of the original sample obtained using MW energy. It is clear from Supplementary Fig. 1 that no significant differences were observed before and after proton beam irradiation.

**Figure 2:**
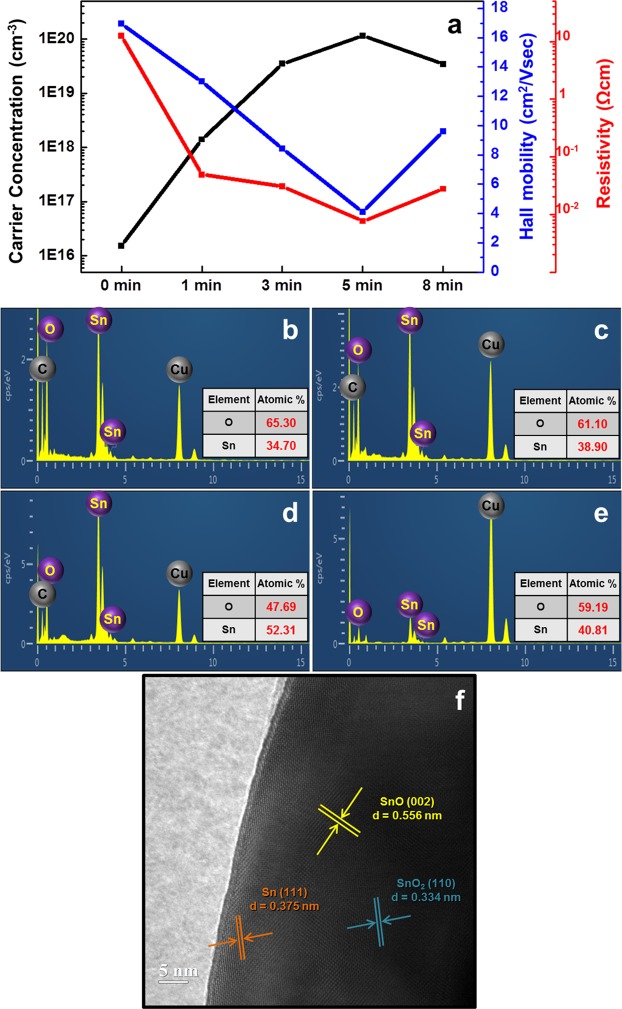
Dependence of electrical characteristics in SnO_2_ thin film and composition of SnO_2_ NWs on microwave energy irradiation time and HRTEM of SnO_2_ NW with 5 min of MW irradiation. (**a**) Carrier concentration, hall mobility, and resistivity of SnO_2_ thin film obtained after microwave (MW) irradiation for 0, 1, 3, 5, and 8 min. (**b**–**e**) Elemental analysis for comparison of the ratio of Sn and O after different MW energy irradiation durations: (**b**) 1 min, (**c**) 3 min, (**d**) 5 min, and (**e**) 8 min. All data shown indicate the best oxidation rate when MW was irradiated for 5 min, regardless of the dimension of the SnO_2_ sample. (**f**) Interplanar spacings of SnO_2_, SnO, Sn with 5 min of MW irradiation.

In order to investigate the difference between the samples in the two conditions that are not distinguished in the SnO_2_ NW state, Fig. 2 indicates (1) the carrier concentration, Hall mobility, and resistivity values of SnO_2_ thin films before and after MW irradiation for different durations (1, 3, 5, and 8 min; Fig. 2a), (2) the point EDX analysis corresponding to MW irradiation at different times (1, 3, 5, and 8 min) in SnO_2_ NWs (Fig. 2b–e, respectively), and (3) the interplanar spacings in the HRTEM of SnO_2_ NW with 5 min of MW irradiation (Fig. 2f). Figure 2a shows the tendency of the electrical characteristics of samples before and after 5 min of MW irradiation and all of the common characteristics of these graphs have a particular critical point; in the case of the carrier concentration, the value increases continuously up to 5 min and gradually decreases thereafter. However, the hole mobility and electrical resistance take their lowest values at 5 min. It can be said that the reduction effect (transition from SnO_2_ to Sn) in the SnO_2_ samples is maximized only for up to 5 min of MW irradiation. This analysis can also be seen in the SnO_2_ NW-sample data shown in Fig. 2b–e, which is the elemental analysis after 1, 3, 5, and 8 min of MW irradiation. This data shows that the ratio of Sn/O at 5 min (Fig. 2d) was higher than that of the other conditions (i.e, 1 min (Fig. 2b), 3 min (Fig. 2c), 8 min (Fig. 2e)). This indicates that the optimum conditions for the reduction of SnO_2_ are the same as those of the data shown in Fig. 2a, and irradiation for 5 min or more may cause the re-oxidation of Sn (i.e., from Sn to SnO_2_) due to the heat generated during the reaction (Fig. 2e). As shown in Fig. 2a,d, the sample with 5 min of MW irradiation is the condition in which SnO_2_ is the most effective reducing reaction. Therefore, even if the reduction to complete Sn does not occur, it is observed that the overall composition eventually show a trend of compositional change from SnO_2_ in the centre to Sn on the surface via SnO_x_. From this point of view, the interplanar spacings measured from the centre to the surface of the sample with 5 min of MW irradiation almost coincide with 0.334 nm of SnO_2_ (JCPDS No. 41–1445), 0.556 nm of SnO (JCPDS No. 13–0111), and 0.375 nm of Sn (JCPDS No. 05–0390), respectively^24,25^ (Fig. 2f). In short, the reduction ratio in all metal oxides necessarily includes a critical point as a function of energy exposure duration, which is a maximum value of reduction, and varies according to the energy supply. Due to the fact that re-oxidation can occur after this point, because of the energy generated in the sample, it is necessary to establish and recognize the criteria for redox processes.

**Figure 3:**
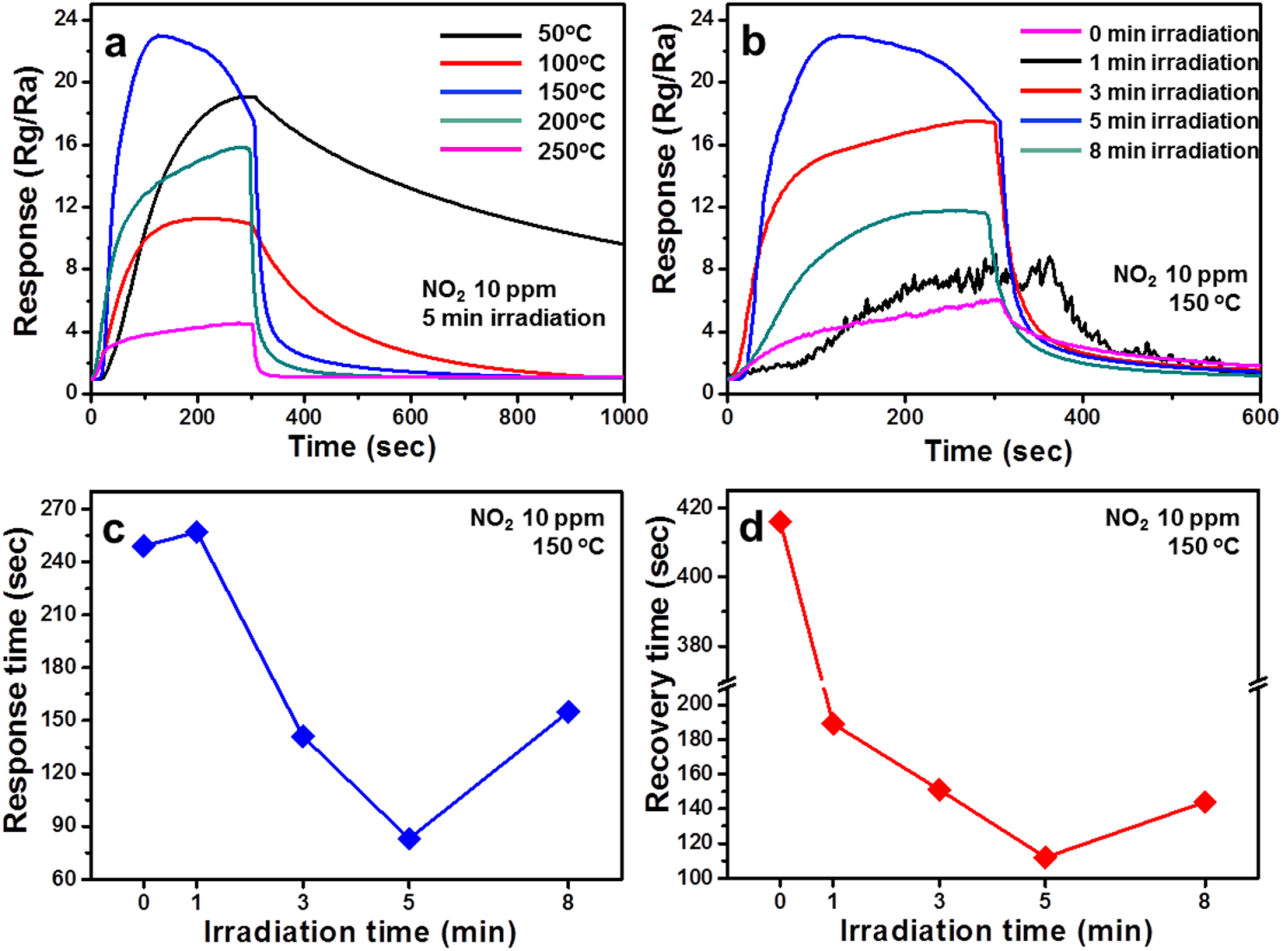
The optimum temperature condition for 10 ppm NO_2_ gas and the characteristic gas sensing graphs for the SnO_2_ NWs produced under different energy conditions. (**a**) NO_2_ gas sensing data of SnO_2_ NWs irradiated with microwave (MW) energy for 5 min, for selected temperatures in the range between 50 and 250 °C; (**b**) NO_2_ gas response at 150 °C in SnO_2_ NWs with different MW energies from 0 to 8 min; (**c**) response time and (**d**) recovery time of SnO_2_ NWs with MW energy for 5 min.

The optimal surface reduction effect of these metal oxides can be used as a gas sensor because the reactivity can be controlled by changing the channelling width according to the reduction degree. First, in the case of gas sensing, it is important to find the optimum gas sensing condition for each temperature because the reactivity is sensitive to temperature^26,27^. Figure 3a shows the 10 ppm-NO_2_-gas characteristics data of SnO_2_-based NWs with MW for 5 min at different temperature ranges (i.e., 50–250 °C). For comparison, in Fig. 3a, we plotted the sensing data for each energy (i.e., 1, 3, 5, and 8 min of MW irradiation), measured with the temperature fixed at 150 °C. In the SnO_2_ NW samples that had received 5 min of MW-irradiation, the largest response (Fig. 3b) and the shortest response time and recovery time (Fig. 3c,d) were measured. Detailed sensing data for different MW-energy conditions from 0 to 8 min are displayed in Supplementary Fig. 2. With regard to the SnO_2_ sample without an energy injection, the maximum gas reactivity occurred at 250 °C (Supplementary Fig. 2a), while the energy-injected SnO_2_ showed its maximum gas reactivity at 150 °C. Furthermore, even if the proton energy instead of MW energy is used, the best gas sensing temperature zone is also 150 °C (Supplementary Fig. 3a,b), which compares favourably to the existing optimal processing temperature of 250 °C for SnO_2_ gas sensing^28,29^. The data in Supplementary Fig. 3a,b is evidence that the effect can be the same if the surface is reduced, regardless of the type of energy. The degree of reduction on the surface of the metal oxide, therefore, is a more critical factor than the type of energy. Moreover, all the sensing data (Fig. 2 and Supplementary Figs 2 and 3) converge as a simple but powerful cause-effect (i.e., cause-consequence), as already mentioned before. Thus, as the degree of reduction from the surface to the centre is continuously varied like gradation effect, the energy barrier between the dissimilar materials (i.e., heterojunction) decreases. Further, as the physical distance of the depletion layer decreases, it can be interpreted that the gas sensing characteristics, such as the response, response time, and recovery time, are highly dependent on the state of the sample. For reference, the response values for the other gases are shown in the Supplementary Fig. 4 under the same condition that the NO_2_ gas exhibits the best sensing characteristics.

**Figure 4:**
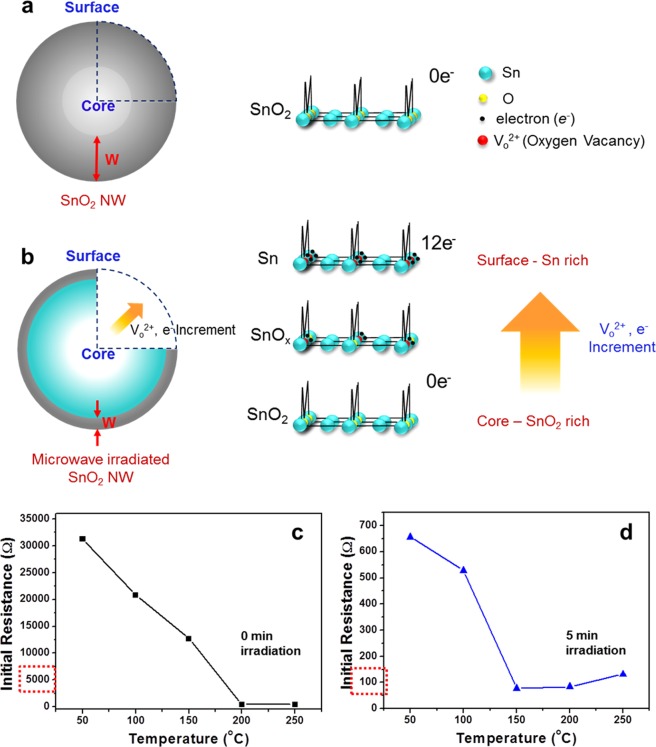
Schematic diagram showing reduction from the surface to the centre of SnO_2_ nanostructures and initial resistance measurements before and after high-energy irradiation. (**a,b**) Comparison of stoichiometric Sn and O ratios in SnO_2_ nanostructures (**a**) before and (**b**) after high-energy irradiation. Theoretically, there is no electron carrier in SnO_2_ before the high-energy irradiation, but after the high energy irradiation, relatively Sn-rich and SnO_2_-rich states occur on the surface and in the centre, respectively; temperature dependences of (**c**) initial resistance of SnO_2_ nanostructures before high-energy irradiation, and (**d**) initial resistance of SnO_2_ nanostructures after high-energy irradiation.

Regardless of the type of sample (one- or two-dimensional) and type of energy irradiated (MW- or proton-energy), the basic concept of metallization of surface reduction (MSR), which we consistently observed in our experiments, is displayed in Fig. 4. When the synthesized SnO_2_ is in an ideal state, the stoichiometric ratio is exactly 1 (Sn): 2 (O), so that the carrier concentration becomes zero (Fig. 4a). However, given sufficient energy, this energy will gradually change the material, starting at the surface and gradually penetrating into the core, causing a continuous gradation effect of Sn (surface)–SnO_*x*_ (between surface and core) –SnO_2_ (core) (Fig. 4b). Thus, this effect can generate a final Sn-rich state at the surface from the initial SnO_2_-rich state, with an excess of electron carriers remaining due to a stoichiometric imbalance originating from the oxygen vacancies at the surface. The metallization of SnO_2_ to Sn on metal oxide surfaces can be easily inferred by comparing (1) the high initial resistance before energy irradiation^30,31^ (Fig. 4c) and (2) the low initial resistance after energy irradiation^32,33^ (Fig. 4d). In particular, the much lower initial resistance (i.e., approximately 100 Ω) after 5 min of energy irradiation means that the reduction efficiency was most prominent for this irradiation duration, suggesting that metallization was the most active for this experimental condition. This metallization of surface reduction technique differs from existing methods in its capacity to modify from the surface, creating the gradation effect, which is analogous to a continuous energy transition in an energy band. Therefore, it will be possible to switch to a material suitable for the needs up to a desired area in a much simpler manner, while overcoming the disadvantages of the current doping technique.

## Discussion

Unlike conventional doping methods, the metallization of surface reduction (MSR) technique not only increases carrier concentrations, but also enables smooth energy connection with the base material. In particular, this method has the advantages that desired results are obtained more easily and with greater stability. The gas sensor measurements have proved that the change of the carrier causes the energy change of the base material and can be represented by a large characteristic change in the application field. It is assumed that the MSR technique is possible not only via MW- and proton-beams as we investigated in this study but also through the use of any possible energy source. Since the MSR technique is based on energetic connections and changes that can affect not only the surface but also the base material, it will therefore provide a breakthrough for existing scientific research lines and new theories.

## Methods

### Synthesis of one-dimensional SnO_2_ nanowires

First, in order to synthesize SnO_2_ nanowires (NWs), 1 g of Sn powder (>99.9%, Sigma-Aldrich) was uniformly dispersed in an alumina boat (100 mm × 20 mm × 15 mm) and the Si substrate (4-inch silicon wafer, Sigma-Aldrich) was placed upside down on the Sn powder in the alumina boat, and the boat was inserted into the centre of a horizontal quartz tube. Then, the quartz tube was heated to 900 °C at a rate of 10 °C/min. A vacuum in the tube was maintained at 10^−3^ Torr using a rotary pump. When the temperature reached 900 °C, 300 sccm of O_2_ gas and 1,700 sccm of Ar gas were injected into the gas tube and maintained for 1 h. The vacuum level of the tube after the gas was injected was maintained at 2 Torr. After 1 h of processing time, the gas was closed and the furnace cooled for 6 h. When the sample was removed from the tube, it was observed that white-coloured SnO_2_ NWs had grown on the Si substrate.

### Fabrication of two-dimensional SnO_2_ thin film

In order to compare the effects of SnO_2_ thin films with those of SnO_2_ nanowires, a uniform SnO_2_ thin film was synthesized on a Si substrate using atomic layer deposition (ALD). The ALD processing temperature was 200 °C, and Tetrakis(dimethylamino)tin (TDMASn; [(CH_3_)_2_N]_4_Sn) and deionised water were used as sources of Sn and O, respectively. An inlet gas purging sequence was repeated for 500 cycles, in which 1 cycle consisted of injection of TDMASn for 1 s, Ar purging for 20 s, injection of deionized water for 1 s, and then Ar purging for 20 s. Under these conditions, the growth rate of the SnO_2_ thin film deposited on the Si in the chamber was approximately 10 nm/cycle. After 100 cycles, the source of Sn and O was closed and the resultant homogeneously deposited SnO_2_ film was observed after the furnace had been cooled for 1 h.

### High energy beam irradiation method

Two types of high-energy beam (power for microwave (MW) model and power for ion-beam model) were used in order to investigate the amount of reduction on the one-dimensional and two-dimensional SnO_2_-based nanostructures. The microwave irradiation was carried out with different durations, 1, 3, 5 and 8 min, while the proton beam was fixed at an energy of 2 MeV, 10^11^ cm^−2^, and the samples with and without high-energy beam irradiation were compared each other.

### Gas sensing conditions for metallization of surface reduction (MSR) effect

For practical applications of SnO_2_ obtained under different conditions, gas sensing for NO_2_ gas was performed. For the gas sensor circuit, Ti (50 nm) was deposited as a buffer layer and Au was deposited as an electrode using a silk screen method. The substrate on which the electrical channels are formed is inserted into a gas chamber capable of controlling the temperature from 50 to 250 °C. At this time, the flow rate of the gas mixed with the dry air and the target gas is fixed at 10 ppm, respectively. Electrical characteristics of the all samples with different gas flow rates were measured as gas-characteristic graphs such as response (*R*_g (targent gas)_/*R*_a (dried air)_), response time, and recovery time, by a Keithley 2400 source meter. In addition, the responses of other gases such as ethanol, acetone, benzene, xylene, H_2_S, NH_3_, SO_2_ were compared under the same sensing conditions.

### Electrical change and microstructure analysis

To investigate morphological properties and crystallographic microstructures of SnO_2_-based nanostructures, a series of scanning electron microscopy (SEM; Hitachi S-4200), X-ray diffraction (XRD; Philips X-pert MRD X-ray diffractometer), and transmission electron microscopy (TEM; JEOL JEM-2010, Japan, 200 kV) were performed; measurements using the more-detailed techniques of high-resolution TEM (HRTEM), selected-area electron diffraction (SAED), and energy-dispersive X-ray (EDX) spectrometry, using attachments of the aforementioned TEM instrument, were also carried out. Electrical changes such as carrier concentration, Hall mobility, and resistivity of the SnO_2_ thin films with different energy injection conditions were detected using Hall measurement methods. The data that support the findings of this study are available from the corresponding author upon reasonable request.

## Supplementary information

Supplementary Information
