# Supplementary material for: New type of doping effect via metallization of surface reduction in SnO2
Source: Sci Rep. 2019 May 31;9:8129. doi: 10.1038/s41598-019-44634-0 (PMC6544616; doi:10.1038/s41598-019-44634-0)
Supplement: Supplementary file 1 — Supplementary Information [file 41598_2019_44634_MOESM1_ESM.docx]

**Supplementary information**

**New type of doping effect via metallization of surface reduction in SnO_2_**

Jae Hoon Bang^1^, Myung Sik Choi^1^, Han Gil Na^1^, Wansik Oum^1^, Sun-Woo Choi^2^, Sang Sub Kim^3,^*, Hyoun Woo Kim^1,4^* & Changhyun Jin^1,4^*

^1^Division of Materials Science and Engineering, Hanyang University, Seoul 04763, Republic of Korea

^2^Department of Materials Science and Engineering, Kangwon National University, Samcheok, 25913, Republic of Korea

^3^Department of Materials Science and Engineering, Inha University, Incheon 402-751, Republic of Korea

^4^The Research Institute of Industrial Science, Hanyang University, Seoul 04763, Republic of Korea

*Correspondence to: sangsub@inha.ac.kr (S. S. Kim), hyounwoo@hanyang.ac.kr (H. W. Kim), chjin0910@gmail.com (C. Jin)


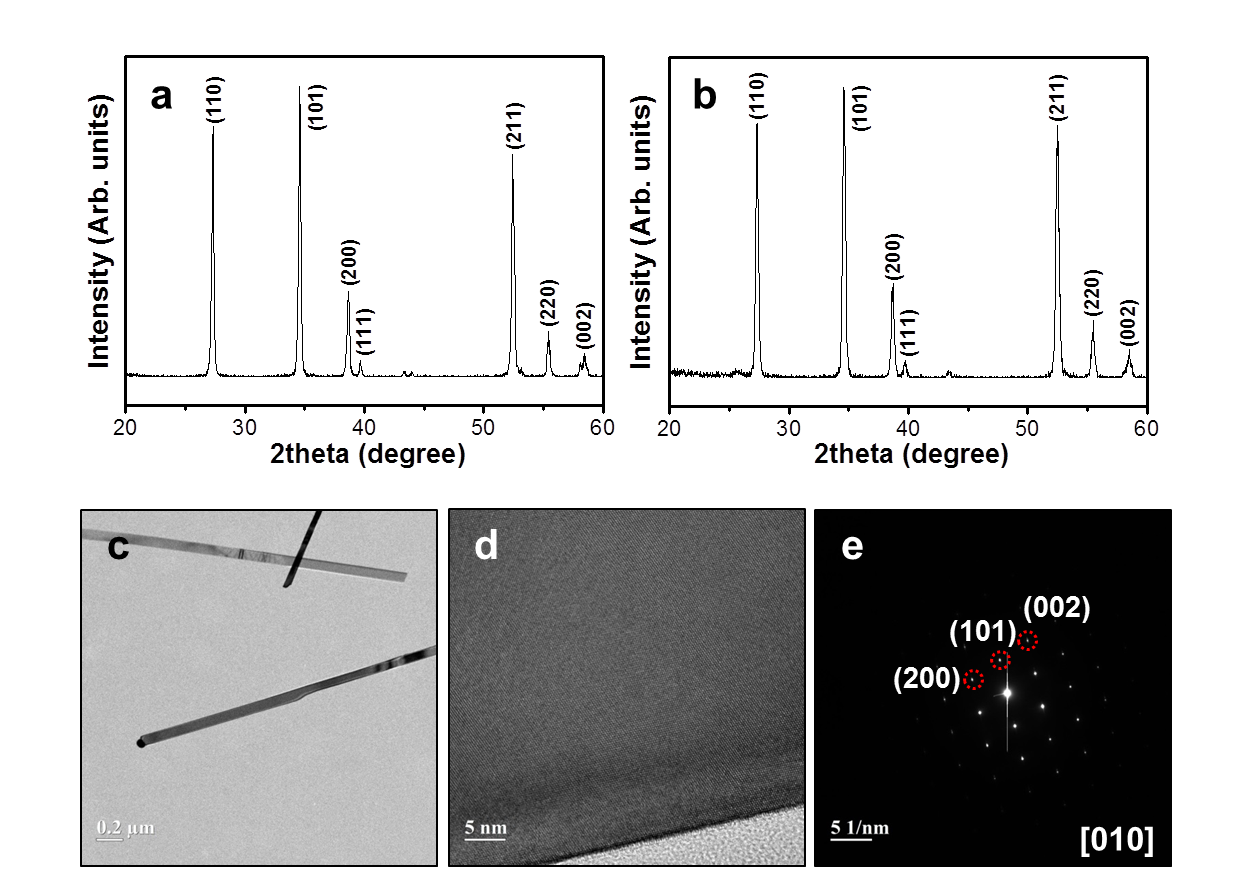


**Supplementary Figure 1 | XRD and TEM analyses of SnO_2_ nanowires using proton energy.** (a–b) XRD (a) before and (b) after proton energy irradiation; (c) low-magnification image, (d) HRTEM, and (e) SAED pattern of SnO_2_ nanowires (NWs) after proton-energy irradiation. At this time, the microstructure and morphology of SnO_2_ NWs seems to be irrelevant to the absence or presence of energy irradiation or its type.


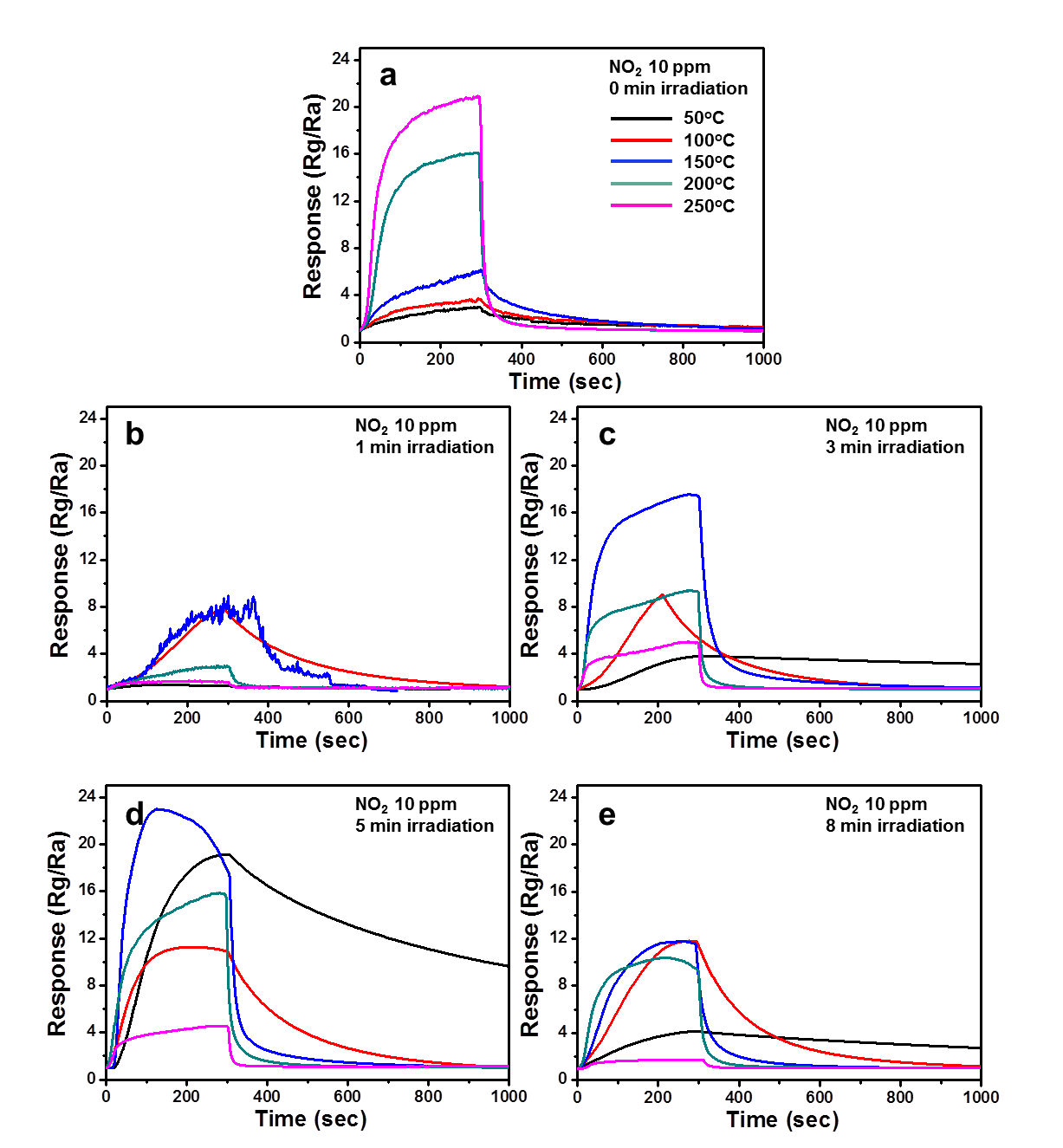


**Supplementary Figure 2 | Comparison of the gas response of SnO_2_ nanowires irradiated for different durations in the temperature range 50–250 °C.** (a–e) NO_2_ gas response of SnO_2_ NWs with (a) no microwave (MW) energy, (b) 1 min, (c) 3 min, (d) 5 min, and (e) 8 min of MW energy, respectively. For the energy irradiated samples (b–e), the best sensing characteristics are exhibited at 150 °C.


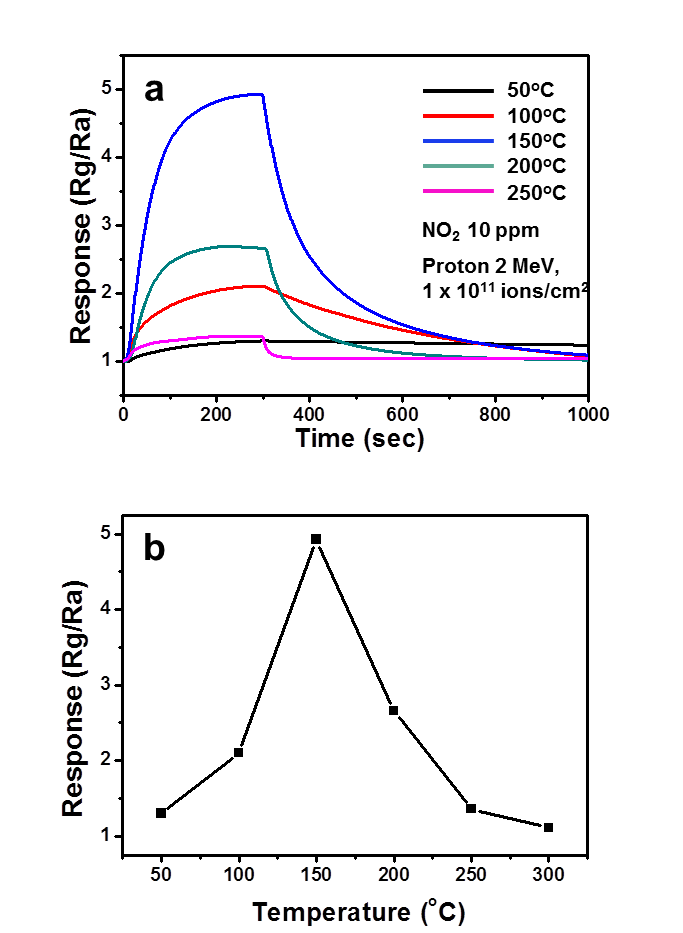


**Supplementary Figure 3 |** Gas sensing data at different temperatures when proton energy is used instead of MW. (a–b) comparison of gas sensing response of SnO_2_ NWs with proton energy between 50 °C and 250 °C.


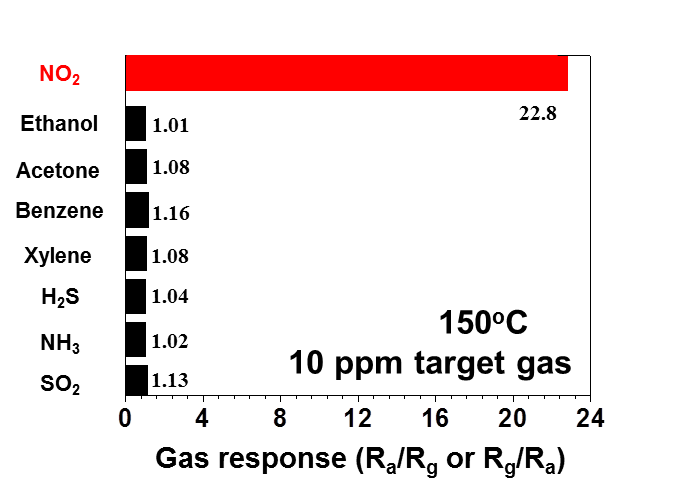


**Supplementary Figure 4 |** Comparison of responses to different gases under the same conditions in SnO_2_ NWs with 5 min of MW irradiation.
